# Multi-Source Information-Based Bearing Fault Diagnosis Using Multi-Branch Selective Fusion Deep Residual Network

**DOI:** 10.3390/s24206581

**Published:** 2024-10-12

**Authors:** Shoucong Xiong, Leping Zhang, Yingxin Yang, Hongdi Zhou, Leilei Zhang

**Affiliations:** 1 School of Energy and Mechanical Engineering, Jiangxi University of Science and Technology, Nanchang 330013, China; xiongsc@jxust.edu.cn (S.X.); yangyingxin@jxust.edu.cn (Y.Y.); 2 School of Mechanical Engineering, Hubei University of Technology, Wuhan 430068, China; zh_hongdi@163.com; 3 Moutai Institute, Renhuai 564507, China; zhangleilei@mtxy.edu.cn

**Keywords:** bearing fault diagnosis, deep learning, residual network, multi-source heterogeneous information

## Abstract

Rolling bearing is the core component of industrial machines, but it is difficult for common single signal source-based fault diagnosis methods to ensure reliable results since sensor signals are vulnerable to the pollution of background noises and the attenuation of transmitted information. Recently, multi-source information-based fault diagnosis methods have become popular, but the information redundancy between multiple signals is a tough problem that will negatively impact the representational capacity of deep learning algorithms and the precision of fault diagnosis methods. Besides that, the characteristics of various signals are actually different, but this problem was usually omitted by researchers, and it has potential to further improve the diagnosing performance by adaptively adjusting the feature extraction process for every input signal source. Aimed at solving the above problems, a novel model for bearing fault diagnosis called multi-branch selective fusion deep residual network is proposed in this paper. The model adopts a multi-branch structure design to enable every input signal source to have a unique feature processing channel, avoiding the information of multiple signal sources blindly coupled by convolution kernels. And in each branch, different convolution kernel sizes are assigned according to the characteristics of every input signal, fully digging the precious fault components on respective information sources. Lastly, the dropout technique is used to randomly throw out some activated neurons, alleviating the redundancy and enhancing the quality of the multiscale features extracted from different signals. The proposed method was experimentally compared with other intelligent methods on two authoritative public bearing datasets, and the experimental results prove the feasibility and superiority of the proposed model.

## 1. Introduction

Rolling bearing is one of the key components in rotating machinery, the failure of which is likely to lead to equipment shutdown and interrupt the normal industrial production process; thus, the accurate fault diagnosis of bearings has important practical significance. Since modern machines belong to a very complex system, date-driven methods are more suitable for rolling bearing monitoring and diagnosis issues [1]. In recent years, plenty of excellent data-driven fault diagnosis methods have been proposed [2].

Traditional data-driven diagnosis methods consist of two processing steps: feature extraction and fault recognition [3]. Machine learning algorithms are capable of building classification models for specific tasks, but they are unable to directly analyze sensor signals due to the lack of adaptive information mining ability. That is why professional researchers need to design the feature extraction step extra carefully. For example, Cheng et al. [4] decomposed the bearing vibration signal by particle swarm optimization variation mode decomposition, calculated and composed the fault feature vector by using the composite multi-scale displacement entropy, and finally input the feature set into the optimized extreme learning machine model for rolling bearing fault diagnosis. Zhang et al. [5] extracted two types of signal features, namely energy entropy and intrinsic mode function matrix singular value, by using integrated empirical mode decomposition and input them into a multi-classification support vector machine optimized for inter-cluster distance through feature space to diagnose the fault type of rolling bearings. Although traditional data-driven approaches have been used in many successful studies, they still have some inherent limitations [6]. First, realistic signals are usually sampled under non-stationary working conditions while being overlapped with heavy background noise, so modern data processing methods require lots of research effort to conquer the information mining issue. Second, traditional bearing fault classifiers are usually shallow machine learning models, which struggle to learn very complex mapping relationships. To tackle the above problems, developing intelligent diagnostic methods with more powerful computing models is necessary and urgent [7].

Deep learning algorithms can automatically realize feature extraction and pattern recognition at one step without the participation of human labor [8]. With the improvement in hardware computing power and the development of training techniques, deep neural networks can become deeper and wider, ensuring stronger representation capabilities. A convolutional neural network is a deep learning variant that is optimized for feature extraction [9] and has been successfully applied in the field of fault diagnosis so far. For instance, Qian et al. [10] proposed a feature transfer learning model based on improved DenseNet and joint distribution adaptation for rolling bearing fault diagnosis. Joint distribution adaptation reduces the marginal distribution difference and conditional distribution difference in learning transferable features, thereby improving the fault diagnosis accuracy of the lightweight DenseNet model. Duan et al. [11] combined the channel attention mechanism, a Siamese deep residual network, and a support vector machine for high-precision bearing health condition recognition. Shao et al. [12] proposed an enhanced deep feature fusion method for rotating machinery fault diagnosis. A new deep auto-encoder is constructed by denoising the auto-encoder and shrinking the auto-encoder, and then the local hold projection is used to fuse the deep features, and finally, the fusion depth features are fed to the softmax function to train an intelligent diagnostic model. Shan et al. [13] proposed a bearing fault diagnosis method based on voiceprint features and Mel-CNN. Variational mode decomposition was utilized to remove the high-frequency component of the motor noise and extract the Mel spectrum voiceprint features, and then the Mel voiceprint features were re-extracted by the Mel-CNN to fully obtain the abstract features characterizing the bearing faults.

However, most of the mainstream rolling bearing intelligent fault diagnosis methods only utilize a single sensor to achieve diagnostic purposes. The single sensing signal is susceptible to background noise interference, information transmission chain attenuation, and other factors, resulting in the signal information not being able to accurately characterize the health status of the monitored objects and misleading the diagnostic model to make wrong judgements. From this perspective, the utilization of multiple sensor signals can achieve more accurate and reliable fault diagnosis [14]. To date, the applications of intelligent rolling bearing fault diagnosis methods based on multi-source information are relatively infrequent, and the information redundancy between multi-source sensing signals is a tough technical problem. To solve these issues, Guan et al. [15] developed a normalized pulse energy kurtosis weighted rule to fuse the multichannel vibration signals and designed a parallel lightweight CNN to achieve feature extraction and classification. Wang et al. [16] analyzed the correlation between the multi-sensor bearing monitoring signals using a similarity measure and fused the multi-dimensional features using a principal component analysis to fully characterize the operation state of rolling bearings. Zhang et al. [17] utilized the residual pyramid algorithm to form two fused acoustic and vibration signals and used two improved 2D-CNNs to extract the fault features contained in the above two signals separately; then, they designed an AdaBoost algorithm with a dynamic deletion mechanism to fuse the features and produce bearing diagnosis results. Wang et al. [18] proposed a multi-source information fusion feature combined with a self-attention mechanism to train the multi-label deep reinforcement learning model for bearing compound fault recognition. Zhong et al. [19] proposed a novel weighted domain adaptation network using residual denoising and multiscale attention for multi-source information-based tunnel boring machine main bearing fault diagnosis, where a deep feature extractor was skillfully designed by incorporating residual denoising and multiscale attention modules, achieving better domain adaptation despite significant domain interference. Zhang et al. [20] combined the multi-head self-attention mechanism with a multi-scale 1D-CNN for monitoring the uneven wear state of high-speed train brake friction blocks; multi-source data were firstly sampled using the Smote-Tomek method, and then the monitoring model comprehensively extracted multiscale features and fused them for diagnosing the wear state. Hu et al. [21] proposed a genetic simulated annealing optimization method with a multi-source data CNN for rolling bearing fault diagnosis, aiming to identify faults more accurately and make full use of multi-source data. Researchers have proposed intelligent bearing fault diagnosis methods based on multi-source information from different angles, but many of them still stayed at the level of shallow fusion of signal features or diagnostic results. The mechanism of deep fusion of multi-source information has not been completely clarified yet, and there is massive untapped potential for developing an intelligent multi-source feature fusion algorithm which could make use of the merits of the automatic adaption of deep neural networks. It is of significance to put forward an efficient solution to this problem.

Aiming to solve the problem of multi-source information-based fault diagnosis, this paper proposes an end-to-end diagnostic model called a multi-branch selective fusion deep residual network (MBSF-DRN). This paper firstly analyzes the merits of multi-source information for rolling bearing fault diagnosis issues and compares the differences between homogeneous information and heterogeneous information. And according to the information characteristics of multi-source signals, the multi-branch network structure of the MBSF-DRN combined with customizable convolution kernel sizes and the dropout technique is specially designed, and it is able to enhance the ability of the deep learning model to extract typical features from the massive and messy information of the original signal and automatically identify faults, effectively alleviating the problem of information redundancy.

The main contributions of this paper are summarized into the following four points:

1. To remedy the limitations of single signal source-based fault diagnosis methods, a new fault diagnosis model called the MBSF-DRN, which can be fed with multiple signals simultaneously, was proposed and successfully achieved satisfactory diagnosing performance.

2. For multi-source heterogenous information, the performance of the MBSF-DRN compared with other similar models shows that independently processing every signal source may be better than simultaneously handling all signals.

3. The multiscale convolution kernel structure of the MBSF-DRN validates that assigning suitable kernel sizes to different input signal sources according to their characteristics is efficient for enhancing the qualities of extracted features.

4. This study proves that dropout technology is effective for solving the problem of multi-source information redundancy.

The rest of this paper is organized as follows: Section 2 details the theories of deep neural networks and demonstrates the structure of a building block in the proposed MBSF-DRN. Section 3 describes the framework of the proposed MBSF-DRN-based fault diagnosis method. Section 4 and Section 5 present the experiments conducted on two rolling bearing datasets to verify the performance of proposed method and discuss several advantages of MBSF-DRN. At last, Section 6 and Section 7 show the discussion and conclusions.

## 2. Theory Background

### 2.1. Convolutional Neural Network

A convolutional neural network (CNN) is a type of neural network that has many advantages over traditional artificial neural networks. Sparse connection and weight sharing are two major advantages of CNNs, which allow for deeper and more complex network architectures. The CNN architecture is composed primarily of three types of layers: a convolutional layer (Conv-layer), pooling layer, and fully connected layer (FC-layer).

The Conv-layer serves as a crucial element in a CNN, utilizing its trainable kernels to generate feature maps by convolving input matrices. Each output neuron is activated by the limited volume of kernels, leading the Conv-layer to extract robust features from small-sized sub-regions of inputs, which are commonly known as local receptive fields. Assuming the input matrix is represented by $x\in R^{\mathrm{mw}\times \mathrm{mh}}$, the kernel by $k\in R^{\mathrm{kw}\times \mathrm{kh}}$, the convolution stride by s∈R, and the output matrix by $a\in R^{\mathrm{nw}\times \mathrm{nh}}$, here, ⌈⋅⌉ is the round-up function, nw=⌈(mw−kw+1)/s⌉, and nh=⌈(mh−kh+1)/s⌉, while the convolution operation in CNN can be defined as follows:

(1) $$\{\begin{array}{l} a=x{*}_{s}k\\ a_{i,j}=\overset{\mathrm{kw}-1}{\underset{p=0}{\sum }}\overset{\mathrm{kh}-1}{\underset{q=0}{\sum }}k_{p,q}x_{is+p,js+q},\forall i,j \end{array},$$

where the symbol “${*}_{s}$” represents the convolution operation with a stride of s.

**Figure 1:**
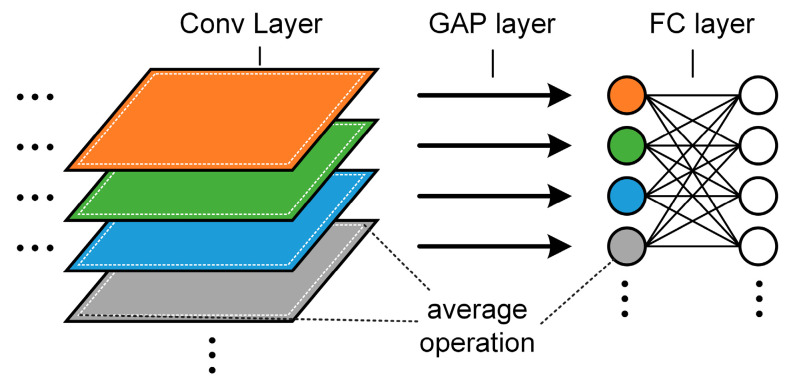
Global average pooling layer.

In this study, there is only one special pooling layer called the global average pooling layer (GAP) included in the proposed network, which served to smoothly connect convolutional layers and fully connected layers. The functioning of the GAP layer is illustrated in Figure 1. GAP has several advantages, such as feature maps that can easily be interpreted as categories of confidence units by enforcing mappings between feature maps and categories. Additionally, by computing the average for each feature map, the model is less vulnerable to the influence of overfitting and can readily adapt to changes in the features’ spatial transformation.

The FC-layer is a classic network layer structure in which each input neuron and output neuron in the layer are interlinked. Given the input vector of $x\in R^{m}$ and the output vector of $a\in R^{n}$, the output of an FC-layer can be calculated as

(2) $$\{\begin{array}{l} a=x\cdot W+b\\ a_{i}=\overset{m-1}{\underset{j=0}{\sum }}x_{j}w_{j,i}+b_{i},\forall i \end{array},$$

where $W\in R^{m\times n}$ is the weight matrix, and $b\in R^{n}$ is the bias vector. Through either the Conv-layer or the FC-layer, the outputs are then passed through the ReLU activation function to introduce nonlinearities into the network, except the uppermost FC-layer of the network is followed by another kind of output function.

For a single-label classification task, the cross-entropy loss function is commonly used as it can significantly improve the convergence speed of the model weights [22]. This loss function measures the difference between the predicted probability distribution and the actual label distribution and adjusts the model parameters accordingly during training to minimize this difference. The formula of the loss function is given below:

(3) $$L\left(p\left(x\right),q\left(x\right)\right)=-\sum_{i=1}^{C}p_{i}\left(x\right)\cdot \log\left(q_{i}\left(x\right)\right),$$

where *C* is the number of classes, and $p_{i}\left(x\right)$ and $q_{i}\left(x\right)$ are the actual and the predicted probability of the input ***x*** belonging to the *i*th class, respectively.

### 2.2. Residual Learning

In neural networks, each neural layer can be thought of as representing an underlying mapping that requires the solver to approximate. As the network depth increases, the complexity of the mappings that need to be learned also increases, making it more difficult for solvers to find the optimal solutions, which hinders the ability of deep neural networks to learn and perform well on complex tasks. As a result, an advanced technique called residual learning was developed to address this issue.

**Figure 2:**
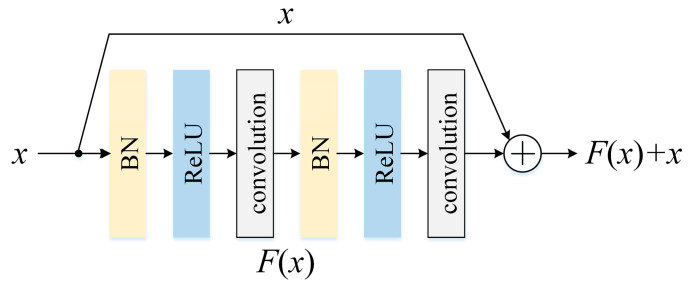
A residual block.

Residual learning [23], also known as skip connections, allows the model to learn only the difference between the actual input and the expected output instead of learning the output itself. This is achieved by adding the input data to the output of one or more hidden layers, as illustrated in Figure 2, which creates a shortcut connection that allows the gradient to flow directly to the previous layers. Residual learning has several outstanding benefits. Firstly, it can help to mitigate the issue of vanishing gradients. Residual connections prompt the network to better retain and utilize information from previous layers, resulting in improved model performance. Additionally, residual learning enjoys a more efficient training process and reduces the risk of overfitting. Finally, since residual connections can be easily added to any kind of neural structure as they provide a flexible approach to designing deep learning architectures that can be customized to different types of data and applications.

In Figure 2, there is a special neural layer called the “BN layer”, which denotes the batch normalization [24] layer. The BN layer helps with gradient flow and speeds up convergence during the optimization process by normalizing the input values for each layer of the neural network. Essentially, the BN layer ensures that the input values are centered and scaled during each training iteration, making it easier for the model to learn more efficiently and accurately. The BN layer has been shown to improve the performance of deep neural networks, reduce overfitting, and make the model less sensitive to the initial values of the weights and biases. For a d-dimensional input $x=\left(x^{1},\ldots ,x^{d}\right)$ over a mini-batch $B=\left\{x_{1},\ldots ,x_{m}\right\}$, the BN layer normalizes the layer inputs as follows:

(4) $$\mu_{B}=\frac{1}{m}\sum_{i=1}^{m}x_{i},$$

(5) $$\sigma_{B}^{2}=\frac{1}{m}\overset{m}{\underset{i=1}{\sum }}{\left(x_{i}-\mu_{B}\right)}^{2},$$

(6) $${\hat{x}}_{i}=\frac{x_{i}-\mu_{B}}{\sqrt{\sigma_{B}^{2}+\varepsilon }},$$

(7) $$y_{i}=\gamma {\hat{x}}_{i}+\beta ,$$

where $\mu_{B}$ and $\sigma_{B}^{2}$ are the expectation and variance of inputs $x_{i}$ over B, respectively, ${\hat{x}}_{i}$ is the normalized input, ε is a tiny constant equal to $1\times 10^{-5}$ in this paper, γ and β are a pair of learnable parameter vectors that scale and shift the normalized inputs elementwise to restore the representation capability of the network layer, and $y_{i}$ is the final output corresponding to $x_{i}$. BN layers are widely used in many network architectures, and they are also prevalent in the model proposed in this study.

### 2.3. Dropout

Dropout [25] is a technique that is proposed to reduce the risk of overfitting and provide a way of approximately combining exponentially many different neural network architectures. The choice of which units to drop is random, and the simplest case is to retain each unit with a fixed probability independent of other units. Let *x* denote the vector of inputs into a neural layer; *y* denotes the vector of outputs from the layer. *W* and *b* are the weights and biases at the layer. The feed-forward operation of a standard neural layer with dropout can be described as

(8) $$r_{j}\sim \mathrm{Bernoulli}\left(p\right),$$

(9) $$\tilde{x}=r*x,$$

(10) $$z_{i}=W_{i}\tilde{x}+b_{i},$$

(11) $$y_{i}=f\left(z_{i}\right),$$

where “∗” denotes an element-wise product, and r is a vector of independent Bernoulli random variables, each of which has a probability, p, of being 1. This vector is sampled and multiplied element-wise with the inputs of that layer x to create the thinned inputs $\tilde{x}$. The thinned inputs are then used for forward calculation in this layer.

In this paper, other than utilizing the regularization and ensemble ability of the dropout technique, the characteristic of randomly removing units from the network structure is also specially favored to help reduce feature information redundancy. Since the layer input *x* becomes thinner after the dropout, which means the amount of the features participating in each training process is reduced, it is believed that abstract features will learn to be more characteristic and corporate with each other instead of interfering. And to make the dropout more sensible, a value dr=1−p is used in the rest of this paper for setting up the dropout rate.

## 3. Proposed MBSF-DRN Method

### 3.1. Discussion about Multi-Source Information

When the rolling bearing is deeply buried inside the machine equipment and the sensor can only be installed on the surface of the bearing housing, the quality of the signal collected by the monitoring system is bound to deteriorate, and it is more difficult to ensure reliability when using the fault diagnosis method based on a single signal. Thus, the fault diagnosis method based on multi-source information has gradually attracted attention and been developed. There are many methods that have combined multiple signal sources for fault diagnosis, among which some combine the same type of signal sources on different parts, and others combine different types of signal sources on the same object. Considering that the fault information patterns of the same type of signal sources are mostly similar, the redundancy of information between signals will be far greater than the difference. Therefore, this paper declares that selecting different sensor types for signal sources, which are also called multi-source heterogeneous information, can enjoy more benefits, thereby achieving better fault diagnosis performance.

There are many different types of monitoring signals for rolling bearings, including vibration, current, voltage, noise, temperature, etc. Each of them contains abundant information that helps to capture various bearing faults. However, due to the influences of sampling modes and the background environment, different signals usually contain different kinds of information compositions. For example, vibration sensors are usually adsorbed on the outer surface of bearing housing, and it is inevitable for the collected vibration signals to contain a lot of environmental noise and the vibration information of other machine parts. Current signals are usually collected by instantly monitoring the working current of the driving motor. Compared to vibration signals, current signals do not worry about the environment noise too much, but there is trouble in the sense that the majority of power consumption comes from the other moving parts and useful information related to the rolling bearing is severely overwhelmed. All in all, the useful fault information is very weak in both vibration signal and current signal, but since each kind of signal has a specific situation, designing concrete data analysis methods for different signal sources is worth considering.

### 3.2. Typical Architecture of MBSF-DRN Model

**Figure 3:**
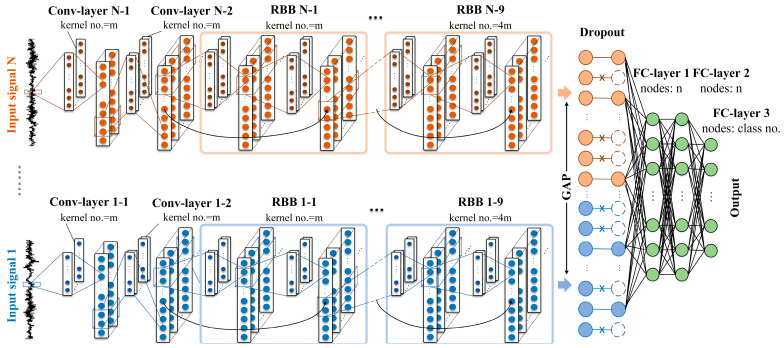
Architecture of MBSF-DRN model. “RBB” denotes the residual building block.

In view of the characteristic differences between multiple signal sources, to fully extract the fault information of each signal and effectively fuse them, a multi-branch selective fusion deep residual network is proposed in this paper. Figure 3 depicts the architecture diagram of the MBSF-DRN, which consists of several network branches and four FC-layers. Every network branch structure is mutually independent, which means no possible network connections or data interactions exist. The MBSF-DRN directly takes some original signals as inputs, and each signal flows into a separate network branch, going through a series of activities of complex and abstract feature extraction. The amount of input signals in the MBSF-DRN can be flexibly set up according to different tasks since any number of branch structure can be freely loaded in the network architecture. But generally, in consideration of the potential saturation of signal information and the expensive cost of monitoring hardware, most multi-source heterogeneous information-based fault diagnosis methods usually just use 2~4 signals.

In each network branch, two 1D Conv-layers followed by L consecutive residual blocks (whose structures are shown in Figure 2) are responsible for processing the specific signal source, and the size of convolution kernels should be designed carefully according to the characters of the input signal. For instance, it is preferable to use longer kernels when processing the vibration signal because the high-frequency regions of vibration signal contain lots of background noise. The features extracted by the long 1D kernels tend to be low-frequency information, thus effectively alleviating the influence of irrelevant noise. Relatively speaking, the current signal also contains a large amount of interference information, but it is not as serious as the vibration signal, so shorter convolution kernels can be selected. The advantage of a shorter convolution kernel is that the frequency domain of the search range of features can be expanded, which is conducive to more fully capturing the fault information of signals.

The purposes and benefits of designing multiple network branches in the architecture of the MSPF-DRN are as follows: The first is to enable allocating different convolution kernels sizes for the feature extraction of different signal sources, as described in the above paragraph. The second is to prevent the abstract features of heterogeneous signals from coupling together. Since convolution kernels fuse the information of multiple channels of the input matrices, a certain correlation relationship between different channels is required [26]. However, heterogeneous signals are usually independent of each other, and even sampling synchronization cannot be guaranteed in some cases, so it is difficult for heterogeneous signals to satisfy the information correlation requirement. If the convolution kernel is extracting features on multiple heterogeneous signals simultaneously, it is bound to experience certain performance degradation due to the forced coupling of features. Alternatively, a deep separable convolution (DSC) kernel [27] can also alleviate the above problem, but the DSC kernel completely gives up the channel information, limiting the diversity of extracted features, so the multi-branch network structure is a better choice.

Following the convolutional network branches, the GAP layer transfers the whole feature maps into category confidence units and then FC-layers make the final decision. In this process, the potentially severe feature redundancy due to the information similarity of different monitoring signals will have a negative effect on the precise decision-making process and waste the model’s representation power. To enhance the representativeness and diversity of the extracted abstract features, the MBSF-DRN uses the dropout technique at the input of the FC-layers. By randomly deleting some feature output responses with a large probability, each time, only parts of abstract features are retained for fault category decision during training; in this way, the model tends to selectively extract and fuse the representative features on each piece of signal information and purposely ignore the features with high dependencies and similarities, and finally, the capacities of the MBSF-DRN model can be fully exploited and further improved.

### 3.3. The Diagnosis Process of the Proposed Framework

**Figure 4:**
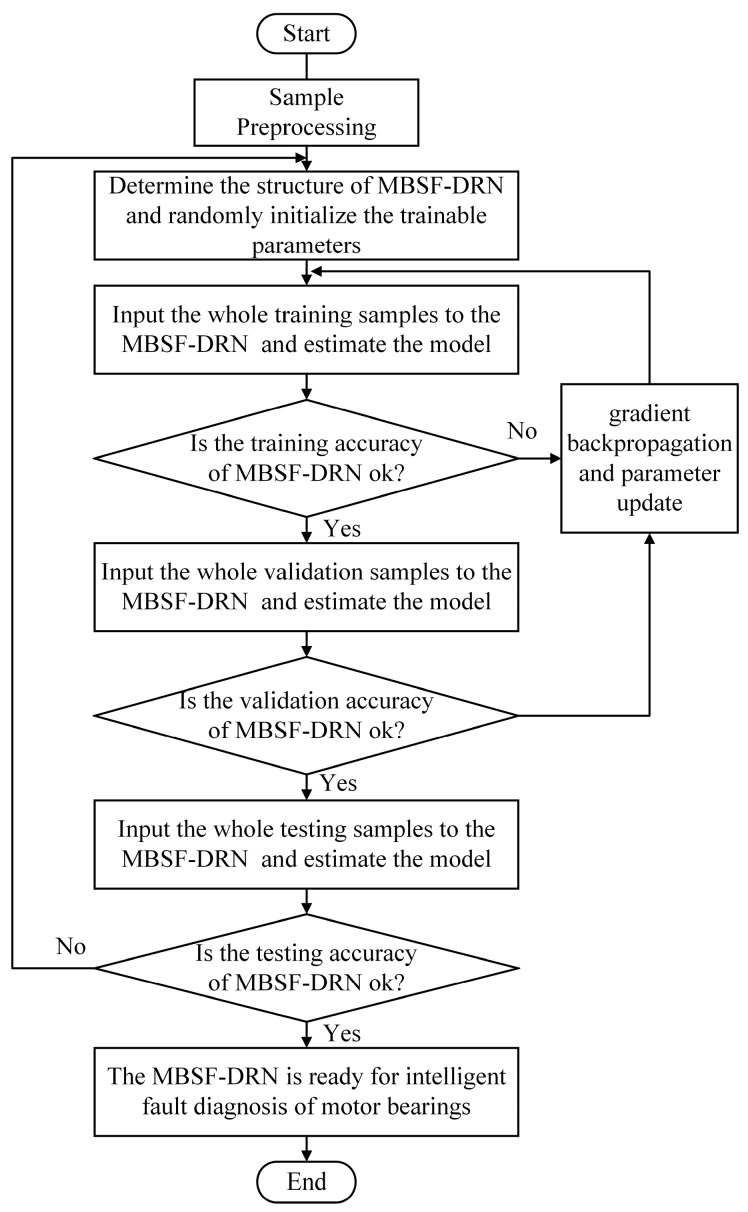
The flowchart of the overall fault diagnosis process of the proposed MBSF-DRN.

In this study, an enhanced fault diagnosis framework based on multi-source heterogeneous information and the MBSF-DRN is proposed to handle complex bearing fault diagnosis tasks. The MBSF-DRN receives original time-domain signals as inputs and efficiently makes full use of precious fault features while designedly alleviating the burden of multi-source information redundancy. The flowchart of the proposed fault diagnosis framework is shown in Figure 4 and summarized as follows:

1. Step 1: Collect different kinds of heterogeneous signals of the rolling bearing under various health states and pre-process the signals with data cleaning, normalization, slicing, labeling, etc. Construct a one-dimensional multichannel signal dataset and divide it into the training, validation, and testing sets.
2. Step 2: Establish the MBSF-DRN model by determining the structure hyper-parameters (including the number of input signal sources and stacked residual blocks, the number and size of convolution kernels, dropout rate, number of fully connected neurons, etc.) and randomly initialize the trainable parameters of the MBSF-DRN model.
3. Step 3: Input the whole training samples to the MBSF-DRN model and perform a forward calculation to obtain the outputs of the model and their errors with the labels.
4. Step 4: Estimate whether the training accuracy of the MBSF-DRN model meets the requirement, and if it is met, perform step 5; otherwise, perform step 6.
5. Step 5: Input the whole validation samples to the MBSF-DRN model to estimate whether the validation accuracy of the MBSF-DRN model meets the requirement, and if it is met, perform step 7; otherwise, return to step 6.
6. Step 6: Perform the gradient backpropagation calculation, obtain the gradient values of each layer of trainable parameters, update the parameters with the pre-defined optimization rules, and return to step 3. The detailed method of training proposed by the MBSF-DRN model can be seen in Algorithm 1.
7. Step 7: Input the whole testing samples to the MBSF-DRN model to estimate whether the testing accuracy of the MBSF-DRN model meets the requirement, and if it is met, perform step 8; otherwise, return to step 2.
8. Step 8: The MBSF-DRN model is ready to perform intelligent fault diagnosis for rolling bearings.

**Algorithm 1.** Algorithm for training MBSF-DRN model.Training MBSF-DRN. We use default values of $l=0.0001,epoch_{max}=20,\;\;m=32,\;\;val\_acc_{thresh}=0.99,\;\;\beta_{1}=0.9,\;\;\beta_{2}=0.99$.**Require**: Initial learning rate l, the maximum of total training epochs $epoch_{max}$ batch size m, total sample number of training dataset $n_{train}$, validation accuracy threshold for early-stop $val\_acc_{thresh}$, Adam hyper-parameters *β*_1_, *β*_2_, MBSF-DRN model $F_{\theta }$. **Require:** initial MBSF-DRN parameters *θ*_0_. 1:  **for** e = 1, … , $epoch_{max}$ **do** 2:    $n=n_{train}$ 3:    while n ≥m **do** 4:      **for** *i* = 1, … , *m* **do** 5:        Sample a data *x* from training dataset. 6:        *n* = *n* − 1 7:        $\tilde{y}\leftarrow F_{\theta }\left(x\right)$ 8:        $L^{\left(i\right)}\leftarrow \mathrm{CrossEntropyLoss}\left(y,\;\;\tilde{y}\right)$ 9:     end for
10:     $\theta \leftarrow \mathrm{Adam}\left(\nabla_{\theta }\frac{1}{m}\overset{m}{\underset{i=1}{\sum }}L^{\left(i\right)},l,\beta_{1},\;\beta_{2}\right)$ 11:    **end while** 12:    Calculate the accuracy of $F_{\theta }$ on the validation dataset. 13:    **if** validation accuracy $\geq val\_acc_{thresh}$ **do** 14:      Stop the training process. 15:    **end if** 16:  **end for**

## 4. Experimental Verification

**Figure 5:**
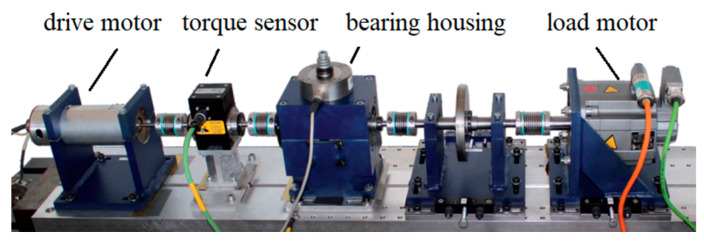
The bearing fault diagnosis test bed.

The authoritative public rolling bearing dataset provided by the University of Pabodeen [28] is used as the experimental sample set for validating our proposed method. The test bed for this bearing dataset is shown in Figure 5, which consists of the drive motor, torque sensor, bearing housing, load motor, and so on.

**Figure 6:**
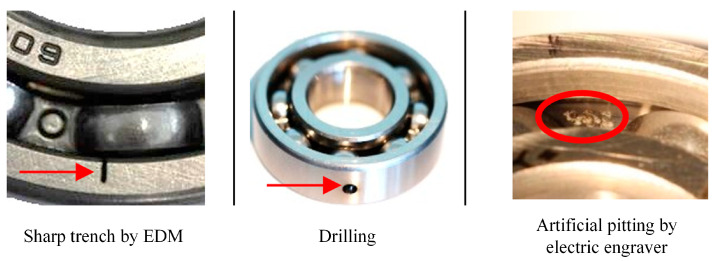
Artificial bearing damages of outer race fault and inner race fault.

The test bearings used in this experiment were the 6203 deep groove ball bearings, the sampling signals included the vibration signals collected at the bearing housing and the current signals collected at the driving motor, both of which had a sampling frequency of 64 kHz. The whole test bearings could be mainly divided into three states: healthy, outer race fault, and inner race fault. Each kind of fault mode was artificially introduced by several processing methods such as drilling, manual electric engraving, and electro-discharge machining, which aimed to simulate the diversity of bearing failure behaviors, as shown in Figure 6. Each fault type had two levels of damage, which are “mild” damage and “moderate” damage, respectively. Specifically, the levels are based on the length of the damage, where “mild” damage denotes that the damage length of the incorporated fault is less than 2 mm, and “moderate” damage denotes that the damage length is between 2 and 4.5 mm. According to the changes in rotating speeds, load torques, and bearing radial forces, there are four different working conditions, $K_{0},\;\;K_{1},\;\;K_{2},\;\mathrm{and}\;K_{3}$, as shown in Table 1. The $K_{0}$ working condition has a maximal rotating speed of 1500 rpm, a 0.7 N × m 379 load torque, and a 1000 N radial force, respectively, and compared with the $K_{0}$ condition, the $K_{1}$ condition decreases the rotating speed to 900 rpm, the $K_{2}$ condition decreases the load torque to 0.1 N×m, and the $K_{3}$ condition decreases the radial force to 400 N. In summary, four working conditions basically approximate the various changes in bearing operation parameters.

In order to prove that the proposed MBSF-DRN method can adaptively conquer the data distribution difference caused by the change in bearing working conditions, the training set, validation set, and testing set of the experimental dataset are collected under different working conditions, that is, the training and validation sets sample data from the $K_{0},\;\;K_{1},\;\mathrm{and}\;K_{2}$ conditions, and the testing set samples data from the $K_{3}$ condition. The test bearings were formally classified into five different bearing states: healthy, mild outer ring fault, moderate outer ring fault, mild inner ring fault, and moderate inner ring fault. For each working condition and each bearing state, one thousand signal segments were randomly sampled from the data file, each signal segment contained two signal channels (vibration and current), and each channel contained 1024 data points. To make the experiment more difficult, every signal had an addition of 3 db of white noise. In summary, the training set and validation set totally contained 15,000 samples (the ratio between the training and validation samples was 9:1), and the testing set contained a total of 5000 samples, and each bearing state was labeled as shown in Table 2.

The raw vibration signal and current signal of the bearing are concurrently input into the MBSF-DRN model for training. Since there are only two kinds of input signal sources, the specific network structure of the MBSF-DRN is shown in Table 3 in which the current signal is input in the first branch, and the vibration signal is input in the second branch. In the MBSF-DRN, every convolutional layer and fully connected layer is followed by a BN layer, and the activation function used was the ReLU function. The output function used was the SoftMax function since this was a single-label fault diagnosis task.

**Figure 7:**
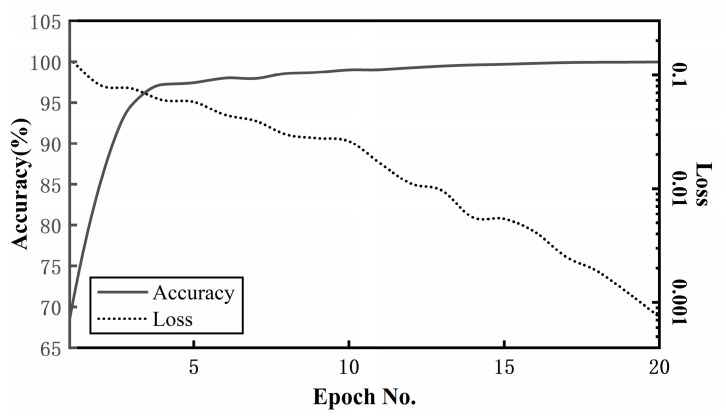
Tendency chart of validation accuracy and loss value of MBSF-DRN in one trial.

The trainable weights of the MBSF-DRN were randomly initialized by resorting to a Gaussian distribution with a standard deviation of $\sqrt{2/n_{l}}$, where $n_{l}$ denotes the weight number of the layer *l*. The Adam [29] optimizer was selected to update the parameters, and $\beta_{1},\;\beta_{2}$ were set to 0.9 and 0.99, respectively, which are two good default values. The learning rate chose a relatively small initial value of 0.0001 since Adam has a fast converging speed. The batch size was set to 32, and the total epoch number was set to 20 to ensure the sufficient convergence of the training loss values. The dropout rate *dr* was set to 0.5. The proposed MBSF-DRN method was implemented in the programming language Python 3.7 with the help of the deep learning library PyTorch 1.11.0 and trained with an Intel^®^Core™i5-7500 CPU and an NVIDIA GTX1080 Ti GPU. The changing processes of the validation accuracy and the loss function value of the MBSF-DRN in one trial are shown in Figure 7.

As can be seen from Figure 7, with the progress of the training process, the classification accuracy of the MBSF-DRN model on the validation dataset steadily increased, and the loss function value gently decreased, and when the training epoch number reached about the 12th epoch, the validation accuracy of the model converged to near 100% and the loss function value went below 0.01. This result shows that the MBSF-DRN was successfully trained on the experimental dataset, which proved its excellent feature mining and classification learning performance.

### 4.1. Performance Comparison between Deep Learning Methods

**Figure 8:**
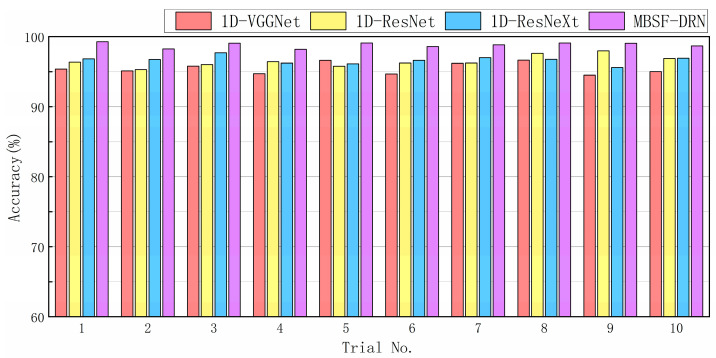
Ten trials of prediction results on experimental testing dataset.

In order to validate the effectiveness of the MBSF-DRN, three modern deep learning models were selected to compare the testing accuracies on this experimental dataset, which are called VGGNet [30], ResNet [23], and ResNeXt [31], respectively. Since the experimental inputs of the deep learning models were 1D time-domain signals, to ensure the equity of comparison, we transform the original VGGNet, ResNet, and ResNeXt models to 1D mode by modifying the whole 3 × 3 2D convolution kernels to 1 × 9 1D convolution kernels. The 1D-VGGNet represented the performance of classical CNN models and had 19 layers with similar kernel weights and amounts of fully connected neurons as the MBSF-DRN. The 1D-ResNet had one Conv-layer followed by nine residual blocks, and three extra FC-layers were added at the top of network structure with no dropout. The weight amount of the 1D-ResNet was also purposely designed so as to be as similar to the weight amount of the MBSF-DRN as possible. The 1D-ResNeXt had a similar structure as 1D-ResNet with the branch number in each residual block set to 2. During the training and testing processes of the 1D-VGGNet, 1D-ResNet, and 1D-ResNeXt, the vibration and current signal were combined into a two-channel composite signal as the model input. All the deep learning models were repetitively trained ten times, and the average prediction accuracy was selected as the measure term. Figure 8 illustrates the testing accuracy results, and Table 4 compares the final statistical results.

From the results, it can be seen that the MBSF-DRN achieved optimal testing results with an average accuracy of 98.82% and a standard deviation of 0.00355, which means it surpassed the three other deep learning models with a satisfactory margin. Compared with the MBSF-DRN, the 1D-VGGNet model has a wonderful CNN model structure and basically represents the performance baseline of modern deep learning models; in this experimental testing dataset, the 1D-VGGNet model ended up with an inferior average accuracy of 95.47% as well as a larger standard deviation of 0.00759, which means the performance of the MBSF-DRN not only improved from the VGGNet-like deeply stacked network structure, but it was also improved due to some extra merits. The difference between the 1D-ResNet and the 1D-VGGNet models is mainly the skip connection structures, and the results prove that residual learning is effective and necessary. The experimental performance of the 1D-ResNet model was a 96.49% average prediction accuracy and 0.00774 standard deviation, which surpassed the 1D-VGGNet model with a 1.02% accuracy gain and added no extra trainable parameters. It is believed that the model capacity of the MBSF-CNN is partly derived from residual learning, but the 1D-ResNet model was still worse than the MBSF-CNN with about a 2.33% accuracy gap. To further evaluate the effect of the multi-branch structure design, the 1D-ResNeXt model was compared with the 1D-ResNet model, and the results prove that the two models had similar prediction performance with only a 0.16% accuracy difference, which may be explained by the fact that only two branches in a residual block is not adequate to fully exert the effect of aggregated transformation. The comparison shows that the two-branch structure design in the MBSF-DRN may also not be able to bring evident capacity benefits. Given the above, it could be verified that the design idea of the MBSF-CNN was efficient for processing multi-source heterogeneous information. Even though other deep learning models like 1D-ResNeXt can automatically fit the experimental datasets to a certain extent without introducing any prior knowledge, they may still be confused by the information nonsense coupling and redundancy problems of multiple signals and deteriorate the prediction performance. The MBSF-DRN takes these factors into consideration and allocates a unique branch for each input signal and adds a dropout for abstract features, resulting in at least a 2.17% accuracy improvement to be freely gained. To summarize, the proposed MBSF-DRN is valid for multi-source heterogeneous information-based fault diagnosis.

Furthermore, to validate that the multiscale kernel size setup of the MBSF-DRN is indeed beneficial for improving feature extraction quality and thus enhancing performance, two other MBSF-DRN variants are tested to compare the accuracy difference. The first variant is called MBSF-DRN-ks25, and both of its input branches use the hyper-parameter setup of Branch 2 in the original MBSF-DRN, that is, two convolutional layers and residual blocks of both branches have kernel sizes of 1 × 49 and 1 × 25, respectively. Similarly, the second variant is called MBSF-DRN-ks9, and both of its input branches use the hyper-parameter setup of Branch 1 in the original MBSF-DRN, that is, two convolutional layers and residual blocks of both branches have kernel sizes of 1 × 25 and 1 × 9, respectively. The two variants follow the same training procedure as the original MBSF-DRN, and the final testing results are shown in Table 5.

In Table 5, it is seen that the two MBSF-DRN variants both have slightly worse performance than the original MBSF-DRN model, indicating that allocating the kernel size according to the characteristic of the input signal is reasonable. Besides that, MBSF-DRN-ks9 and MBSF-DRN-ks25 both attain better performance than 1D-ResNeXt, showing that independently extracting features from every input signal other than simultaneously handling all signals is also effective, which alleviates the complexity while enhancing the efficiency of feature extraction work.

### 4.2. Comparison between Different Dropout Rates

**Figure 9:**
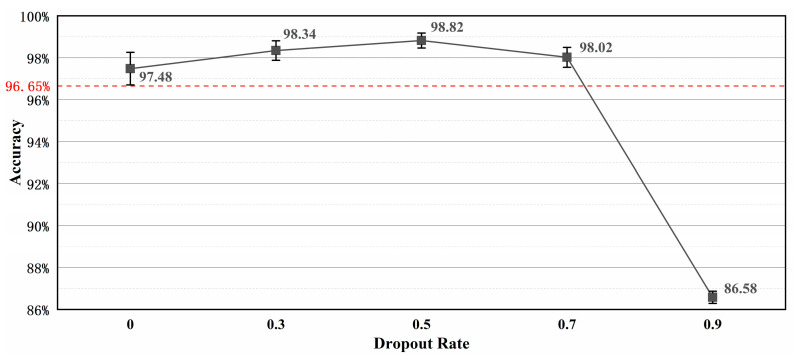
Comparison results of the MBSF-DRN between different dropout rate values.

In the above section, the hyper-parameter of the dropout rate, dr, is set to 0.5 without a given explanation, but actually, dr = 0.5 is a suitable choice to balance the feature uniqueness and the model capacity problem. To further explain the necessity of setting up an appropriate dropout rate value to fully exert model performance, in this section, the hyper-parameter dr is set to 0, 0.3, 0.5, 0.7, and 0.9, respectively, to compare the model testing results. Except for the dr, the other hyper-parameter remained unchanged during the whole comparative experiments. After running ten repeated trials for each mode setup for the dr, the average testing accuracies on the experimental dataset were obtained and are shown in Figure 9.

Figure 9 shows that the model accuracy did not keep improving as the dropout rate increased. It can be seen that when the dr was equal to 0, the average testing accuracy of the MBSF-DRN was 97.48%, which surpassed the 96.65% average accuracy of 1D-ResNeXt with 0.83%. This result proved that the MBSF-DRN did not have trouble with coupling irrelevant features, so it performed better than the other original deep learning models. And when the dr increased from 0 to 0.3, the dropout technique started to play a role in improving model performance, and the average accuracy of the MBSF-DRN smoothly improved from 97.48% to 98.34%. Furthermore, as the dr further increased from 0.3 to 0.5, the best average accuracy of the MBSF-DRN kept improving to 98.82%. At this time, every fully connected input neuron had a 50% chance of being discarded during each training update, which was an intuitively reasonable setting since there are two kinds of signal sources providing double-valued fault information. And the dropout technique deleted potentially redundant information, properly avoiding interference between abstract features. But after the dr increased from 0.5 to 0.7, as the neuron discarding possibility became higher, the model capacity of the MBSF-DRN started to be negatively influenced by largely reduced feature information, and the average accuracy decreased from 98.82% to 98.02%. Even further, when the dr increased to 0.9, the MBSF-DRN became hard to converge, and the final average testing accuracy heavily deteriorated to 86.58%. These results illustrate that the balance between redundancy elimination and model capacity is a significant consideration in assigning suitable dropout rate values. In summary, the proper setup of the dr should be equal to 0.5 for this experiment.

### 4.3. Comparison between Multi-Source Heterogeneous Information and Single-Source Information

**Figure 10:**
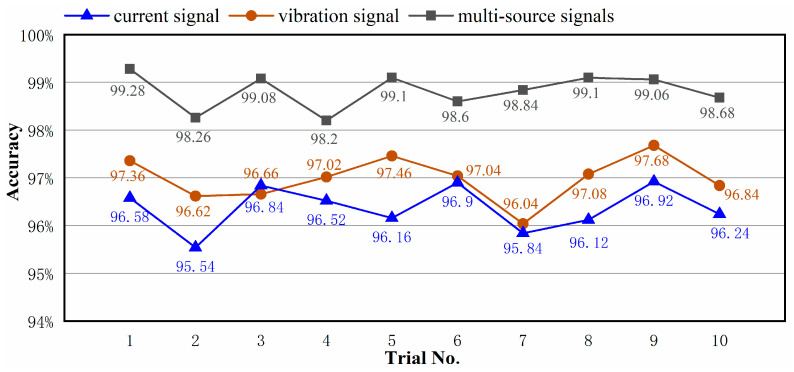
Comparison results of the MBSF-DRN between different signal sources.

The MBSF-DRN in this experiment uses both vibration signal and current signal as inputs to avoid the misjudgment easily caused by single sensing data. The key features of heterogeneous information from multiple sources can complement each other, which is conducive to improving the fault diagnosis performance. In this section, to verify the effectiveness and superiority of multi-source information fusion, the MBSF-DRN was inputted with a single vibration signal, single current signal, and multi-source heterogeneous signals, respectively, for training, and the same model structure and optimizing schedules as the above section were used. In the case that the input signal source only included the vibration signal or current signal, the signals were simultaneously sent to Branch 1 and Branch 2 of the MBSF-DRN for feature extraction. The training process was repeated ten times for each situation, and the results are presented in Figure 10.

From the results in Figure 10, it is seen that feeding the MBSF-DRN with multi-source heterogeneous signals produces a remarkable performance improvement. Its maximal accuracy is 99.28%, the minimal accuracy is 98.20%, and the mean accuracy is 98.82%. In comparison, feeding the MBSF-DRN with a single vibration signal source obtains a maximal accuracy of 97.68%, a minimal accuracy of 96.04%, and a mean accuracy of 96.98%. Inputting single current signal sources obtains a maximal accuracy of 96.92%, a minimal accuracy of 95.54%, and a mean accuracy of 96.37%. The results also show that the vibration signal has slightly better information quantity than the current signal, and the MBSF-DRN effectively fused the multi-source heterogeneous information, taking the prediction accuracy to a higher level.

**Figure 11:**
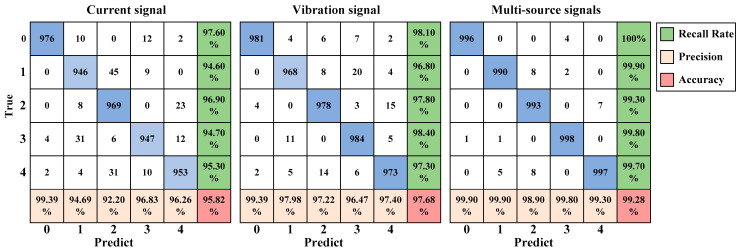
The confusion matrix of the MBSF-DRN model trained on different signal sources.

**Figure 12:**
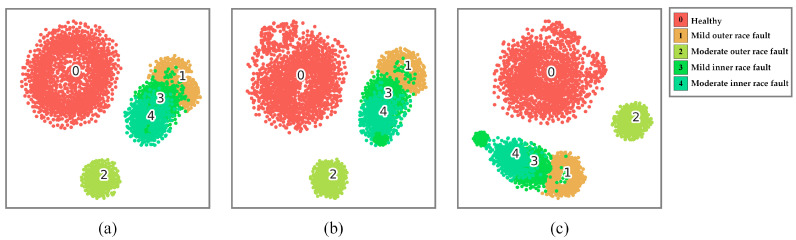
Feature distributions of the MBSF-DRN model at the output of the dropout layer trained on (**a**) a current signal, (**b**) vibration signal, and (**c**) multi-source signals.

Figure 11 presents the confusion matrices of the MBSF-DRN’s testing accuracy under three different input signal sources, among which the recall rate measures how many positive samples are correctly predicted and precision measures how many predicted samples are correctly positive samples. And Figure 12 demonstrates the feature distribution conditions of the three models in Figure 11 at the output of the dropout layer using t-SNE technology. Through comprehensive observations of the recall rates, precisions, and accuracies, it is found that the MBSF-DRN with a single current signal source may have some difficulty in classifying samples in labels 1, 3, and 4. The MBSF-DRN with a single vibration signal source may be a little weak in classifying samples in labels 1 and 4. Additionally, the MBSF-DRN with multi-source heterogeneous signals took advantage of the whole input information, well classifying the samples for every health state, so that the recall rate and precision were always high even for the samples that were hard to distinguish in label 1 and 4. The results prove that using multi-source heterogeneous information is valid for rolling bearing fault diagnosis tasks.

## 5. Industrial Application

**Figure 13:**
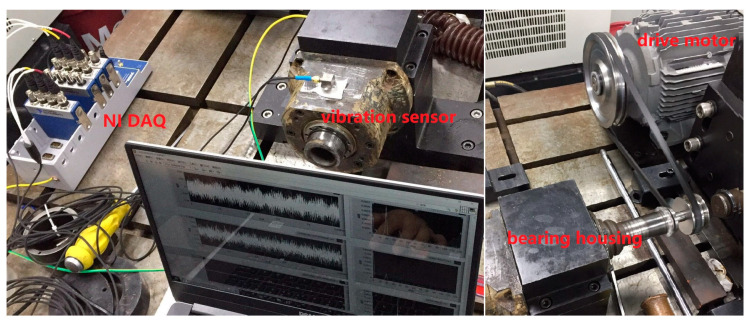
The bearing experimental platform for simulating industrial applications.

To verify that the proposed MBSF-DRN method can also obtain satisfying performance in industrial applications, a bearing fault dataset collected from an experimental platform that mimics the actual failures of rolling bearings in industrial scenarios, as shown in Figure 13, is applied to evaluate the model in this section.

### 5.1. Data Description

There were two kinds of signal sources in this experiment: the vibration signal and the current signal. Vibration signals were sampled at 25.6 kHz using the three-directional vibration sensor, and only signals on the Z-direction were applied. Current signals were also collected at 25.6 kHz, and the W-phase current of the drive motor was used for diagnosis. To simulate rolling bearing damages in realistic cases, tungsten steel powder was artificially incorporated into lubricating grease to pollute the bearing health state. According to the volume of incorporated tungsten steel powder, four bearing states were designed, as shown in Table 6. The NSK 40BNR10H bearings in different conditions were rotating at various speeds, and two signals for each rotating speed were recorded simultaneously.

After acquiring the signals, the training set and the testing set separately drew samples from different rotating speeds. For classes 0, 1, and 2, the training set was sampled under the rotating speeds of 4800 and 5100 rpm, each of which sampled 1500 observations; the testing set was sampled under speeds of 3000, 3600, and 4200 rpm, each of which sampled 1000 observations. For class 3, the training set sampled 3000 observations under the rotating speed of 669 rpm, and the testing set sampled 3000 observations under the speed of 393 rpm. In sum, there was a total of 12,000 samples both in the training set and the testing set. The validation set was generated by randomly picking out 1200 samples from the training set. Each sample contains two signal channels (the vibration signal channel and the current signal channel), and each channel has 1024 data points.

### 5.2. Fault Diagnosis Result Comparison

In this case, the 1D-VGGNet, 1D-ResNet, 1D-ResNeXt, and the MBSF-DRN models were compared in terms of testing performance, and the model structure and hyper-parameter settings were kept the same as in the above section. Since the amount of input signal is also two in this case, setting the dropout rate value to 0.5 seemed reasonable. To formally verify this viewpoint, the situations with dropout rates equal to 0, 0.3, and 0.7 were also tested. After going through the same training procedure, the final results of all deep learning models are presented in Table 7.

Table 7 shows that the MBSF-DRN achieved the best average testing accuracy compared to the other three deep learning models. From the results of comparing different dropout rate values, it is seen that the dr equal to 0.5 obtained the best performance as expected, and setting the dr to 0.3 or 0.7 obtained slightly worse accuracy than the situation with the dr equal to 0.5. Because the training set in this experiment was smaller and the testing set was larger than the experiments in Section 4, the differences in the testing accuracy between every model were less obvious, but the MBSF-DRN still achieved a 98.75% average testing accuracy and the best testing accuracy of 99.58%, surpassing the other three deep learning models by at least 1.40% and 1.32%, respectively.

**Figure 14:**
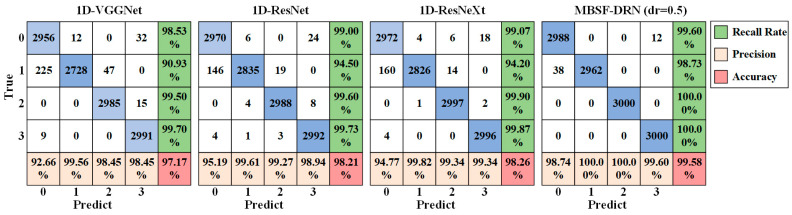
Confusion matrices of the best trials of different models on the simulated industrial dataset.

To more clearly demonstrate the comparison results of every model, Figure 14 presents the confusion matrix of the best trial of 1D-VGGNet, 1D-ResNet, 1D-ResNeXt, and MBSF-DRN in this experiment. It can be seen that the samples of class 1 were the hardest to distinguish, and the majority of misclassifying situations of every model was concentrated on the second row of confusion matrices, that is, the class 1 samples were easy to wrongly classify into class 0 and class 2. This made sense since class 1 showed that the potential failures of the bearings were very slight and hard to observe, and the failure degree was so blurry, so it was inevitable that the models may classify some samples of class 1 into other classes. Even so, the MBSF-DRN showed stronger distinguishing ability between samples and only misclassified 38 samples of class 1 into class 0, while the other three models misclassified hundreds of samples as class 1.

## 6. Discussion

The MBSF-DRN’s performance was compared with that of the 1D-VGGNet, the 1D-ResNet, and the 1D-ResNeXt model on a public bearing dataset and a simulated industrial dataset, respectively. On the first dataset, the MBSF-DRN achieved an average accuracy of 98.82%, which freely surpassed the best counterpart model 1D-ResNeXt model with at least a 2.17% improvement. On the second dataset, the MBSF-DRN achieved a maximal accuracy of 99.58% and an average accuracy of 98.75%, surpassing the other DL models by at least 1.40% and 1.32%, respectively. The two testing results both show the effectiveness of the design idea of the MBSF-DRN model structure since 1D-VGGNet, 1D-ResNet, 1D-ResNeXt, and MBSF-DRN have similar parameter amounts. To be specific, 1D-ResNeXt and the MBSF-DRN both have multi-branch structures, but the performance superiority of the MBSF-DRN verified the advantages of combining independent input processing branches with multiple feature extraction scales. And testing the accuracy changes in the MBSF-DRN between different dropout rate values also verified that the information redundancy problem can be alleviated by dropout technology, but the dropout ratio should be set up carefully.

In future work, to expand the main idea of this paper, we will continually study developing the network structure and finding a method to take the place of the dropout to alleviate multi-source information redundancy more efficiently.

## 7. Conclusions

This study proposes the MBSF-DRN model for multi-source heterogeneous information-based bearing fault diagnosis. The main characteristic of the model is to allocate an independent network branch to each input signal to go through a series of activities of feature extraction, and the shape of kernels could be designed separately according to the characteristics of the input signals. The dropout technique is utilized to remove redundant feature information and enhance the uniqueness of the abstract feature, efficiently improving the model’s predicting performance. The proposed MBSF-DRN was tested on two rolling bearing datasets, and both outperformed the other three original deep learning models. These results show the potential of the MBSF-DRN and inspire the idea of elaboratively designing network structures for enhancing model fault diagnosis performance.

## Figures and Tables

**Table 1:** Operation condition details of test bearings.

Name	Rotating Speed/rpm	Load Torque/N×m	Radial Force/N
*K*_0_	1500	0.7	1000
*K*_1_	900	0.7	1000
*K*_2_	1500	0.1	1000
*K*_3_	1500	0.7	400

**Table 2:** The details of the experimental dataset samples.

Label	Bearing States	Training/Validation/Testing
0	Healthy	2700/300/1000
1	Mild outer race fault	2700/300/1000
2	Moderate outer race fault	2700/300/1000
3	Mild inner race fault	2700/300/1000
4	Moderate inner race fault	2700/300/1000

**Table 3:** The detailed structure of the MBSF-DRN for the experimental dataset.

Structure	Network Layer	Kernel Size	Kernel Number	Stride
Branch 1	Conv-layer1-1	1 × 25	8	2
	Conv-layer1-2	1 × 25	8	2
	*RBB1-1	1 × 9	8	1
	RBB1-2	1 × 9	8	1
	RBB1-3	1 × 9	8	1
	RBB1-4	1 × 9	16	2
	RBB1-5	1 × 9	16	1
	RBB1-6	1 × 9	16	1
	RBB1-7	1 × 9	32	2
	RBB1-8	1 × 9	32	1
	RBB1-9	1 × 9	32	1
Branch 2	Conv-layer2-1	1 × 49	8	2
	Conv-layer2-2	1 × 49	8	2
	RBB2-1	1 × 25	8	1
	RBB2-2	1 × 25	8	1
	RBB2-3	1 × 25	8	1
	RBB2-4	1 × 25	16	2
	RBB2-5	1 × 25	16	1
	RBB2-6	1 × 25	16	1
	RBB2-7	1 × 25	32	2
	RBB2-8	1 × 25	32	1
	RBB2-9	1 × 25	32	1
Tail		GAP layer	/	
		Dropout layer	Dropout Rate (dr): 0.5	
		FC-layer1	Nodes: 64	
		FC-layer2	Nodes: 64	
		FC-layer3	Nodes: 5	

**Table 4:** Statistical results of different models on experimental testing dataset.

Statistics	1D-VGGNet	1D-ResNet	1D-ResNeXt	MBSF-DRN
Max	96.64%	97.98%	97.72%	99.28%
Min	94.52%	95.30%	95.60%	98.20%
Average	95.47%	96.49%	96.65%	98.82%
Std	0.00759	0.00774	0.00544	0.00355

**Table 5:** Testing results of different MBSF-DRN variants.

Statistics	MBSF-DRN Original	MBSF-DRN-ks25	MBSF-DRN-ks9
Max	99.28%	98.78%	98.86%
Min	98.20%	96.82%	97.32%
Average	98.82%	97.89%	98.14%
Std	0.00355	0.00529	0.00618

**Table 6:** Descriptions about four designed bearing health states.

Label	Condition	Description	Rotating Speed (rpm)
0	Normal	Healthy bearings	3000; 3600; 4200; 4800; 5100;
1	Slight damage	No evident phenomenon	3000; 3600; 4200; 4800; 5100;
2	Moderate damage	Slightly overheat	3000; 3600; 4200; 4800; 5100;
3	Heavy damage	Overheat and abnormal sound	393; 669

**Table 7:** The testing results of different models on the simulated industrial dataset.

Statistics	1D-VGGNet	1D-ResNet	1D-ResNeXt	MBSF-DRN (dr = 0)	MBSF-DRN (dr = 0.3)	MBSF-DRN (dr = 0.5)	MBSF-DRN (dr = 0.7)
Max	97.17%	98.21%	98.26%	98.45%	99.13%	99.58%	99.11%
Min	95.88%	96.07%	96.11%	96.80%	97.54%	97.52%	97.78%
Average	96.50%	97.33%	97.35%	97.64%	98.25%	98.75%	98.47%
Std	0.004595	0.007103	0.007724	0.006045	0.005707	0.006302	0.005025

## Data Availability

The original experimental data presented in this study are openly available at https://mb.uni-paderborn.de/en/kat/research/kat-datacenter/bearing-datacenter/data-sets-and-download, accessed on 1 February 2024.
